# Rapid Lymphatic Clearance After Subcutaneous Extravasation of ^177^Lu-PSMA-I&T Radioligand Therapy

**DOI:** 10.1097/RLU.0000000000006595

**Published:** 2026-06-29

**Authors:** Ruben K. Fakkert, Bastiaan J. van Nierop, Rob van Rooij, Marnix G.E.H. Lam, Bart de Keizer

**Affiliations:** Department of Nuclear Medicine and Radiology, UMC Utrecht, Utrecht, The Netherlands

**Keywords:** extravasation, ^177^Lu-PSMA, radioligand therapy, SPECT/CT, dosimetry

## Abstract

Extravasation of therapeutic radiopharmaceuticals during radioligand therapy is uncommon. We report the first published case of subcutaneous extravasation of ^177^Lu-PSMA-I&T, presenting with acute upper-arm swelling, pain, and transient autonomic symptoms. Early planar scintigraphy and SPECT/CT demonstrated intense radiotracer accumulation in the left upper arm, with rapid redistribution and near-complete clearance by 20 hours. Dosimetric analysis estimated an absorbed dose of ~8 Gy at the injection site. Conservative management resulted in no radiation-induced tissue injury. The clinical course was comparable to previously reported cases of ^177^Lu-PSMA-617 extravasation. This case highlights the value of early imaging in the case of extravasation for evaluating the extent of the extravasation and enabling absorbed dose estimation.

**FIGURE 1:**
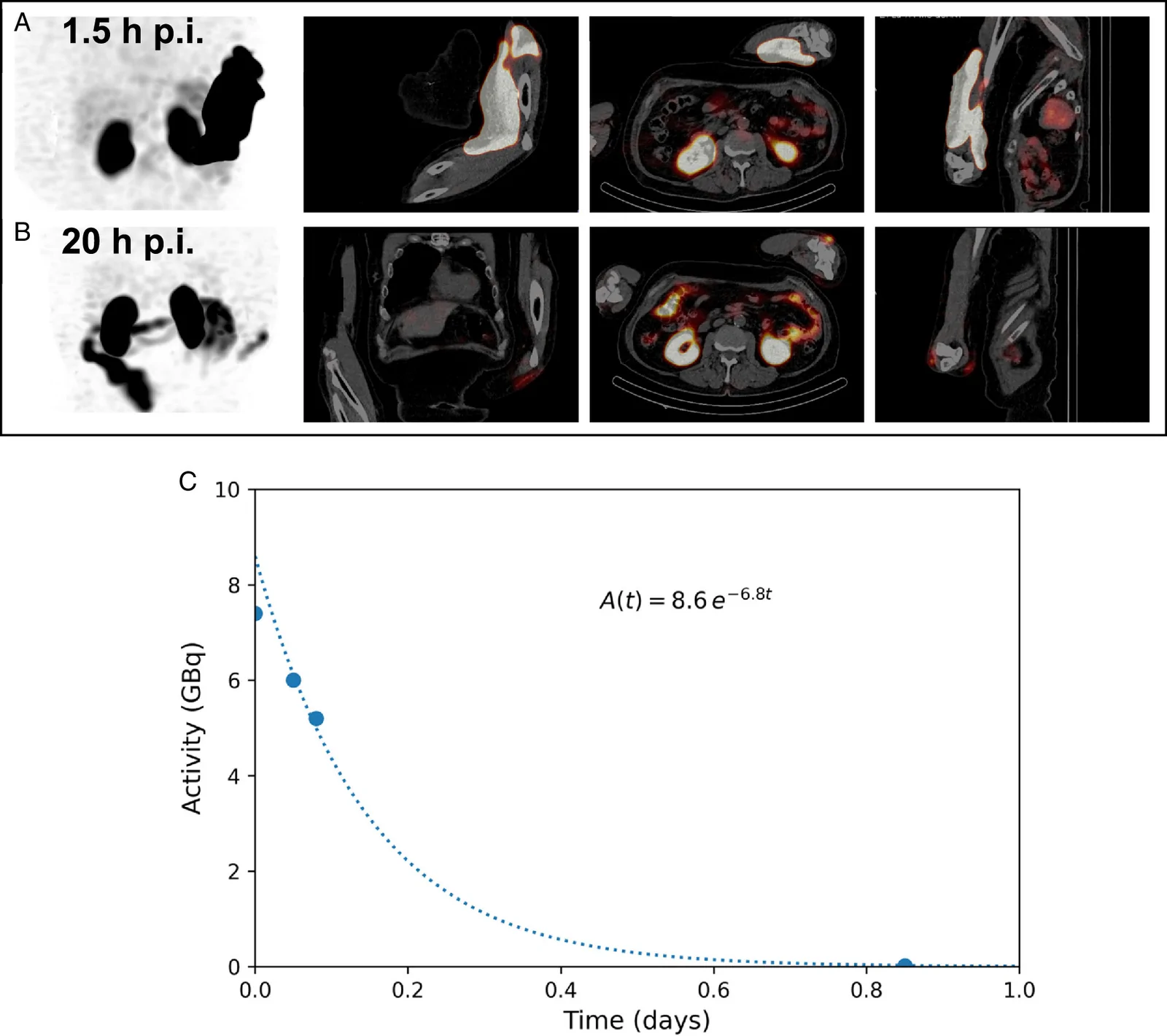
A 78-year-old man with progressive metastatic castration-resistant prostate cancer (mCRPC) was treated with 2 cycles of ^177^Lu-PSMA-I&T radioligand therapy (7.4 GBq every 6 wk).^1,2^ This resulted in a marked biochemical response, with prostate-specific antigen (PSA) levels decreasing from 160 to 0.8 µg/L. ^177^Lu-PSMA-I&T was administered intravenously via a peripheral venous catheter in the left antecubital fossa using an automated syringe pump (RAD-INJECT, Tema Sinergie) over ~1 minute, followed by a saline flush (2×3 mL) at the same flow rate, resulting in a total administration time of ~2 minutes. During the third administration, the patient developed acute swelling and pain of the left upper arm, accompanied by nausea and transient autonomic symptoms. Subcutaneous extravasation at the left elbow was suspected. Physical examination demonstrated diffuse swelling without neurologic deficits or skin compromise. The catheter was removed, the arm was elevated, and cold application was briefly applied for 10–15 minutes. The radiation safety officer was notified in accordance with recommended procedures.^3^ Planar scintigraphy performed at 1.5- and 2 hours postinjection (p.i.) demonstrated intense activity in the left upper arm, corresponding to ~6 GBq at 1.5 hours and 5.2 GBq at 2 hours, consistent with quantitative SPECT findings obtained at ~1.75 hours p.i. (A). As the patient had not voided before imaging, relative quantification was feasible by comparing counts within a region of interest (ROI) around the injection site with total-body counts, enabling estimation of the activity at the injection site (MBq) based on the administered activity. Given clinical improvement, with residual arm swelling and decreasing activity on imaging, the patient was discharged 6 hours after administration in accordance with standard protocol. Follow-up planar scintigraphy with SPECT/CT at 20 hours showed near-complete clearance of activity from the left elbow, with residual activity of ~0.02 GBq (B). Similar early accumulation patterns with rapid clearance (>95% within 24 h) were reported following extravasation of ^177^Lu-PSMA-617, ^177^Lu-DOTATATE, and ^177^Lu-HA-DOTATATE.^4–7^ The absorbed total dose at the injection site was estimated at ~8 Gy using a simplified energy deposition model (275 g soft tissue; β^−^ energy 0.133 MeV/disintegration) (C). Prior reports of ^177^Lu extravasation suggest that absorbed doses up to 10 Gy are generally not associated with clinically significant radiation injury.^6,7^ However, radiation-induced skin toxicity following ^177^Lu-PSMA extravasation has been reported, including delayed pruritus and erythematosquamous skin changes with residual hyperpigmentation.^8^ In this case, the rapid decline in local activity indicated a low risk for deterministic skin effects. Given the relatively small molecular size and low molecular weight of PSMA ligands, these compounds show rapid lymphatic clearance following extravasation, thereby limiting effective half-life and absorbed dose.^3^ No clinical or biochemical toxicities occurred, and PSA further declined to 0.10 µg/L.
